# Extraction of High Value Products from *Zingiber officinale* Roscoe (Ginger) and Utilization of Residual Biomass

**DOI:** 10.3390/molecules29040871

**Published:** 2024-02-16

**Authors:** Alexandra Spyrou, Marcelle G. F. Batista, Marcos L. Corazza, Maria Papadaki, Maria Antonopoulou

**Affiliations:** 1 Department of Sustainable Agriculture, University of Patras, Seferi 2, GR30131 Agrinio, Greece; spyrou.a@upatras.gr; 2 Department of Chemical Engineering, Federal University of Parana, Curitiba CEP 81531-990, PR, Brazil; marcelleguth@ufpr.br (M.G.F.B.); corazza@ufpr.br (M.L.C.); 3 Department of Agriculture, Nea Ktiria, University of Patras, GR30200 Messolonghi, Greece

**Keywords:** ginger valorization, supercritical extraction, subcritical extraction, antioxidant capacity, chemical composition, circular economy

## Abstract

*Zingiber officinale* Roscoe (ginger) is a plant from the *Zingiberaceae* family, and its extracts have been found to contain several compounds with beneficial bioactivities. Nowadays, the use of environmentally friendly and sustainable extraction methods has attracted considerable interest. The main objective of this study was to evaluate subcritical propane (scPropane), supercritical CO_2_ (scCO_2_), and supercritical CO_2_ with ethanol (scCO_2_ + EtOH) as co-solvent methods for the extraction of high value products from ginger. In addition, the reuse/recycling of the secondary biomass in a second extraction as a part of the circular economy was evaluated. Both the primary and the secondary biomass led to high yield percentages, ranging from 1.23% to 6.42%. The highest yield was observed in the scCO_2_ + EtOH, with biomass prior used to scCO_2_ extraction. All extracts presented with high similarities as far as their total phenolic contents, antioxidant capacity, and chemical composition. The most abundant compounds, identified by the two different gas chromatography-mass spectrometry (GC-MS) systems present, were a-zingiberene, β- sesquiphellandrene, a-farnesene, β-bisabolene, zingerone, gingerol, a-curcumene, and γ-muurolene. Interestingly, the reuse/recycling of the secondary biomass was found to be promising, as the extracts showed high antioxidant capacity and consisted of significant amounts of compounds with beneficial properties.

## 1. Introduction

*Zingiber officinale* Roscoe (ginger) is a plant that is widely distributed worldwide and belongs to the *Zingiberaceae* family. It contains compounds that present a wide range of benefits, such as antioxidant, antifungal, antibacterial, and anticancer effects [1,2,3,4]. Ginger, both fresh and dried, is used for consumption as well as medicinal applications [5]. Ginger’s extracts and essential oils have diverse chemical compositions, and they mainly include compounds belonging to terpenoids and phenolics groups which possesses significant biological activity [6]. Moreover, polysaccharides have also been reported to be components of ginger extracts [1]. The bioactive compounds present in ginger extracts/oils lead to a vast number of applications in the medicinal and cosmetic sector, as well as the food industry and the agriculture sector [7,8,9,10,11].

As a plant with significant value, ginger has attracted intensive research interest. Thus, many studies have focused on the application of different extraction methods with the aim of maximizing the extraction efficiency [1]. In the past years, new technologies for the extraction of beneficial compounds have attracted a lot of attention. Specifically, environmentally friendly, low-cost, and sustainable extraction methods have been used to extract and isolate compounds of interest [6]. Among them, super- and sub- critical extraction methods have aroused remarkable interest from the scientific community, because only small changes in the pressure and temperature can increase the selectivity of the extraction process [12]. These types of extractions have numerus advantages, with the most interesting being the decrease of the thermal degradation of the targeted compounds [12], which is a major disadvantage of the conventional extraction procedures [13,14]. The lower temperatures also lead to the reduction of the extraction cost [15].

In the literature, it is well-documented that compounds extracted from ginger possess various biological activities and can have diverse applications. However, the extraction of bioactive compounds with high recovery remains a challenge [5]. Ginger solvent extraction has been the most studied. More recently, advanced methods such as supercritical and subcritical techniques, as well as pressurized fluid and ultrasound-assisted techniques, have shown promising results for the extraction of bioactive substances [5,16]. The evaluation of these techniques for the recovery of such compounds has led to significant interest [5]. It is worth noticing that these types of extractions lead to better quality products, and that the solvent can be recycled and reused [17,18].

The aim of the present study was to explore different methods of ginger extraction, using more advanced and environmentally friendly procedures. Therefore, a variety of sub- and supercritical extractions were performed and evaluated in terms of the yield percentage, the total phenolic content, the antioxidant capacity, and the chemical composition. The gas chromatography-mass spectrometry (GC-MS) technique was also used to identify the main components. Additionally, the utilization of the residual (secondary) biomass was studied, with the purpose being the production of additional added value products. Large amounts of residual biomass, which is potentially not environmentally friendly, are produced yearly [19,20]. There is increasing interest around the utilization of this type of biomass to further isolate natural bioactive compounds [21]. To the authors’ best knowledge, this is the first study focusing on the extraction of ginger through using and comparing a variety of sub- and supercritical extractions. The main focus of the present study was to evaluate the possibility of enhancing the high value bioactive compounds’ recovery through the utilizion of the residual biomass, with a multistep extraction procedure of the secondary biomass. The recovery of those high value compounds is a key point for the valorization of the used biomass, with the possibility of their use in food, cosmetics, and other products. The main limitation regarding the reuse of biomass is the high cost. The proposed procedure is a low-cost, environmentally friendly extraction method that follows the framework of circular economy.

## 2. Results and Discussion

### 2.1. Soxhlet Extraction

**Figure 1:**
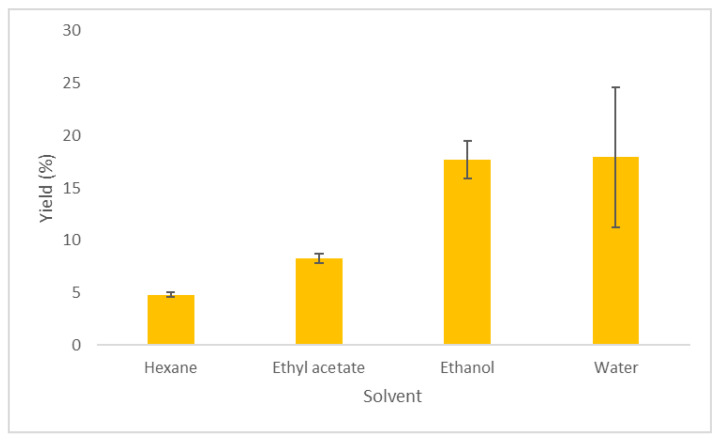
Extraction yield % of *Zingiber officinale* Roscoe using Soxhlet extraction with different solvents.

In Table 1, the extraction conditions and extraction yields using Soxhlet, in combination with different solvents, are presented. The Soxhlet extraction was performed to select the most suitable co-solvent in the supercritical extraction. The highest yield was observed using water as solvent, followed by ethanol (17.93% and 17.70%, respectively) (Table 1, Figure 1). Lower extraction yields were noticed with ethyl acetate and hexane (8.28% and 4.82%, respectively). This difference could be due to the different polarities of the used solvents (10.2 for water, 5.2 for ethanol, 4.3 for ethyl acetate, and 0.0 for hexane) [22], and their ability to solubilize the oils from the biomass.

Similar to our results, Al-Areer et al. found that, [24] when performing Soxhlet extractions at 90 °C for 9 h, the highest extraction yield was observed when ethanol was used as solvent, followed by ethyl acetate and hexane. A different trend was observed by Lemma and Egza, [25] who noticed a higher yield in the hexane than the ethanolic extract when Soxhlet extractions were performed for about 4 h at the boiling point of each solvent.

Moreover, the antioxidant capacity, as well as the concentration of total phenols of the extracts, were evaluated. The results are shown in Table 2. The highest antioxidant activities were observed in the ethyl acetate and ethanolic extracts. Regarding the total phenolic content, the ethanolic extract presented the highest concentration.

The differences in the extraction yields, total phenolic contents, and antioxidant activity may occur because different solvents can extract different active compounds [26]. Water, even though it presented the higher extraction yield, showed the lowest total phenolic content and antioxidant activity. This can be due to the high temperature used in that case (100.5 °C), leading to the thermal decomposition of high bioactive compounds (Table 2). It is well-known that the high temperatures used in the Soxhlet extraction process (up to the boiling point of each solvent), along with the long extraction time, can lead to the thermal degradation of some compounds [27]. More specifically, the continuous evaporation and extraction of the target compounds caused by the solvents, the high extraction kinetics, and the prolonged extraction time can promote the decomposition of thermolabile target compounds [28]. Thermolabile target compounds can consist of polyphenols that present high antioxidant capacity [29].

Μoreover, it is reported in the literature that many factors can affect antioxidant activities. Besides the amount and strength of the antioxidant compounds, the ability of the antioxidants to transfer a hydrogen to a free radical such as DPPH· can also be affected by the environment where the reaction takes place; for example, in different types of solvents [30].

Taking all of the above into consideration, along with the antioxidant capacity (Table 2, Figure S1) of each extract and their total phenolic content (Table 2), ethanol was selected as the co-solvent in the supercritical extraction.

### 2.2. Sub- and Supercritical Extraction

**Figure 2:**
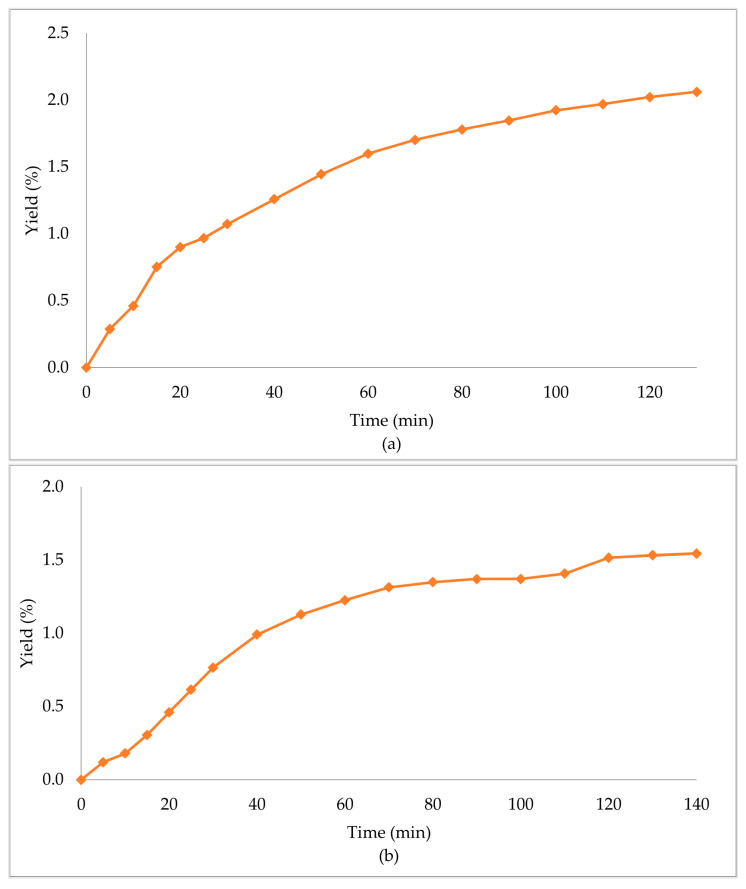

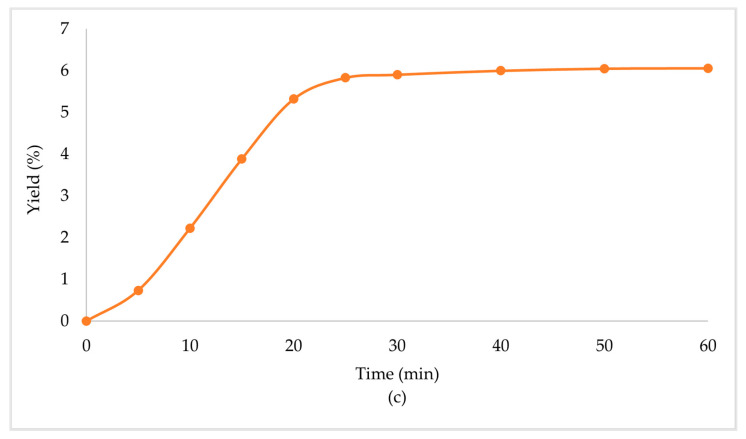
Extraction yield % of *Zingiber officinale* Roscoe extracts acquired under (**a**) scPropane extraction, (**b**) scCO_2_ and (**c**) scCO_2_ +EtOH.

In Table 3, the extraction conditions for the sub- and supercritical extractions are presented. The primary biomass extraction resulted in high yield percentages (Table 4, Figure 2). The supercritical CO_2_ extraction with ethanol as the co-solvent (scCO_2_ + EtOH) gave an extraction yield of 6.06%, which was the highest of the three obtained, followed by subcritical propane (scPropane) and supercritical CO_2_ (scCO_2_), with yield percentages of 2.06% and 1.54%, respectively. It is worth noticing that scCO_2_ + EtOH led to higher yields in less time (half the time in comparison with the other methods). Considering the increase in the yield percentage, and the decrease of the time needed, the addition of ethanol had a positive effect in the overall extraction.

Ethanol, when used as a co-solvent, solubilized the CO_2_ and led to a decrease of the viscosity of the mixture of solvents (CO_2_ + EtOH), and an increase of density [31,32]. The combination of the solvents accelerated the extraction process, led to less usage of CO_,_ and, and enhanced the extraction yield. Additionally, the polar mixture of solvents led to an increase of the extracted amount of polar and soluble compounds [22]. In general, the usage of co-solvents may improve the extraction performance due to the enhanced transport of solute [33].

**Figure 3:**
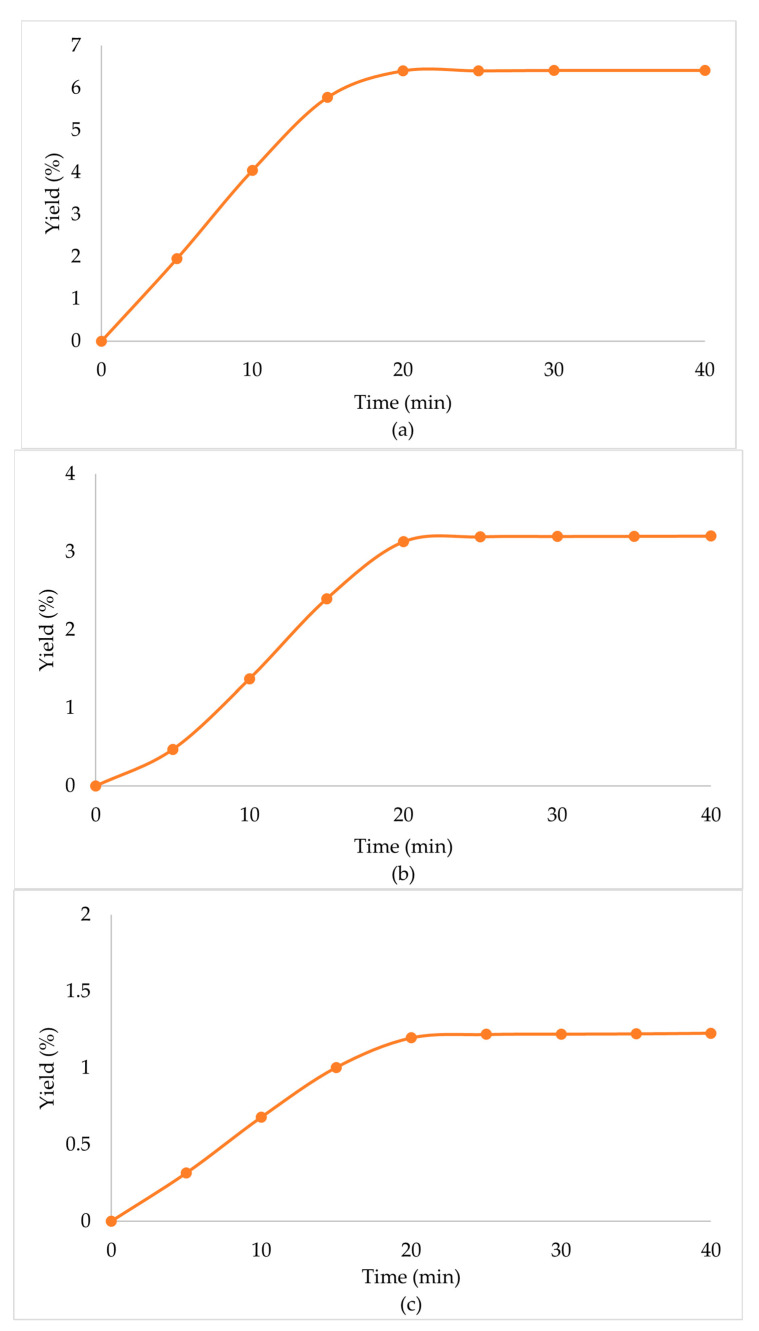
Extraction yield % of *Zingiber officinale* Roscoe extracts acquired under scCO_2_ +EtOH, with secondary biomass, previously used at (**a**) scCO_2_ once, (**b**) scCO_2_ + EtOH once and (**c**) scCO_2_ + EtOH twice.

Due to the enhanced performance of the scCO_2_ + EtOH extraction, when a secondary biomass was used, high extraction yields were observed in all cases (Table 4, Figure 3). Specifically, in the case of the scCO_2_ + EtOH extraction of the secondary biomass that was used once before in scCO_2_ extraction, the extraction yield was significantly higher than that of the primary biomass. CO_2_ behaves as a nonpolar solvent and presents with a low extraction performance for polar compounds [34]. The co-solvent (EtOH) that was used in the secondary biomass extraction had a positive influence on the extraction procedure, since it lowered the viscosity of the solvents, increased the density, and altered the overall polarity of the mixture of solvents, which led to the enhancement of the extraction yield.

When the biomass, prior used in scCO_2_ + EtOH, was extracted in a multistep extraction twice with scCO_2_ + EtOH, the extraction yield was lower than the primary biomass, but still significant. Mainly, this was observed due to the high amounts of polar compounds that the mixture of solvents obtained through the first extraction, and when only CO_2_ was left in the chamber, the capability to extract polar compounds was minimized. In continuation, when the extraction procedure was repeated, and the mixture of solvents renewed, their capability to extract those compounds improved once again and the extraction yield remained significant.

High yield percentages for ginger have been reported by Mesomo et al., [15] and Mesomo et al., [11] using CO_2_ and Propane as solvents, in various pressures and temperatures. For CO_2_, the yield percentages ranged from 0.22% to 3.21%, and for Propane from 1.98% to 2.70%. For CO_2_, the pressure led to a positive effect on the yield, and for propane, both pressure and temperature. Similarly, Salea et al., [35] using scCO_2_ in various conditions, calculated a range of yields from 1.55% to 2.95%. That range was attributed to the variety of pressure and temperature conditions used in each extraction.

Zancan et al. [36] performed scCO_2_ and scCO_2_ + EtOH, and did not observe significant differences in the yield percentages between the two methods, but ethanol favored the extraction of gingerols and shogaols.

### 2.3. Total Phenolic Contents and Antioxidant Capacity

Table 4 presents the total phenolic contents acquired through using different extraction conditions and solvents, as well as both primary and secondary biomass. The total phenolic contents were expressed as mg of gallic acid equivalent per 100 g of sample. In both the primary and secondary biomass, similar total phenolic contents were observed, with the highest being in scCO_2_ + EtOH using secondary biomass, which was formerly used in the same type of extraction.

Stoilova et al., [37] found a total phenolic content of 871 mg/g dry extract acquired through high pressure CO_2_ extraction.

A similar trend can be observed for the antioxidant capacity of the extracts (Table 5, Figures S2 and S3). Overall, according to the IC_50_ values, the scCO_2_ + EtOH extract presented the highest antioxidant activity, and the scCO_2_ extract the lowest.

The antioxidant properties of ginger extracts obtained by different extraction methods have been widely studied. Its antioxidant properties are due to compounds such as gingerols, flavonoids, and phenolic acids [38].

Mesomo et al., [15] studying different conditions of supercritical extraction, observed the highest antioxidant capacity with scCO_2_. Zancan et al. [36] observed that, when no gingerols and shogaols were yet obtained by the extraction the antioxidant, activity was much lower. Stoilova et al. [37] calculated the IC_50_ for inhibition of DPPH to be 0.64 μg mL^−1^.

### 2.4. GC-MS Analysis

Ginger includes both volatiles such as geraniol, borneol, terpineol, curcumene, zingiberol, α-farnesene, α-sesquiphellandrene, α- β-bisabolene, β-elemene etc., as well as non-volatile compounds such as gingerols, shogaols, zingerone, and paradols [7]. The composition of ginger extracts and oils differ significantly. The bioactive compounds of ginger essential oils are mainly monoterpenes and sesquiterpene hydrocarbons, and their chemical composition depends on the nature (fresh/dry) and place of origin of the ginger rhizome, as well as the extraction method employed [10]. They are also composed of oxygenated hydrocarbon compounds including aldehydes, phenols, esters, oxide ethers, alcohols, and ketones [10].

The chemical composition of the extracts/oils was determined by GC-MS analysis, and all the compounds identified have been compiled and presented in Table 6 along with their area percentage. The extracts obtained with all the tested systems were found to have quite similar chemical compositions, and the main substances were a-zingiberene, β-sesquiphellandrene, a-farnesene, β-bisabolene, zingerone, gingerol, a-curcumene, and γ-muurolene. Similar compounds have also been identified in the literature [11,38,39,40]. In the case of scPropane, some more compounds, i.e., monoterpenes and sesquiterpenes, were identified but only in traces. When a primary biomass was used, Soxhlet with ethanol extracted less compounds in comparison with scPropane, scCO_2_, and scCO_2_ + EtOH, signifying the increased efficiency of such advanced extraction techniques.

Our results regarding the main components are in agreement with the bibliography [11]. Based on literature data, higher essential oil and β-zingiberene contents were obtained for the dried ginger rhizome than that of the fresh ones. Moreover, the drying method has been found to play a significant role in essential oil’s yield and its chemical composition. Temperatures lower than 70 °C can increase the yield of ginger oil without having any effect on the transformation of 6-gingerol to 6-shogaol [41].

It is noteworthy that most of the above-mentioned compounds were identified after the reuse/recycling of the secondary biomass, highlighting the possibility to extract the maximum value from the used biomass. In Table 7, the identified compounds of the secondary biomass extractions are presented with their area percentage. After the first extraction of the secondary biomass, the compounds identified were less in comparison with the primary biomass’s identified compounds, but their area percentage was higher.

The main identified compounds (a-zingiberene, β-sesquiphellandrene, a-farnesene, β-bisabolene, zingerone, gingerol, and a-curcumene) that can be found in almost all of the studied extracts have been previously reported to possess significant antioxidant properties. This is in agreement with our results [42,43,44,45,46,47]. Badrunanto et al., [45] when studying the antioxidant components of Indonesian ginger essential oil, observed that, amongst others, a-zingiberene, β-sesquiphellandrene, a-farnesene, β-bisabolene, and a-curcumene, presented a high correlation with the antioxidant activity of the oil. Similarly, Misharina et al. [46] highlighted the antioxidant activity of zingiberene, β-sesquiphellandrene and β-bisabolene. Gingerol analogues have been associated with ginger extracts’ antioxidant activity [42,43,47]. Specifically, Danwilai et al. [42] studied the antioxidant activity of ginger extract oral supplements in cancer patients who were receiving adjuvant chemotherapy. These supplements contained 20 mg day^−1^ 6-gingerol, and results found a significant increase regarding antioxidant activity, and a decrease of oxidative marker levels. Moreover, Wang et al. [43] observed that 10-gingerol presented with about 34.2% DPPH radical scavenging activity, and 6-gingerol about 16.3%. Furthermore, they noticed that the antioxidant activity of those ginger compounds contributed to the antimicrobial activity against *Acinobacter baummannii* infections. 6-gingerol, 8-gingerol, and 10-gingerol’s antioxidant activity was studied by Dugasani et al. [47], who observed that the DPPH scavenging potential was in the order of 10-gingerol > 8-gingerol > 6-gingerol. Rajan et al. [44] studied zingerone’s antioxidant activity using a DPPH free radical method, and observed a dose dependent increase of the compound’s antioxidant activity.

In general, it is well reported in the literature that the components of ginger extracts/essential oils have significant bioactivities and health-promoting properties, and thus can have applications in various sectors [7,8,9,10,11]. Based on literature data, ginger extracts/oils containing most of the bioactive compounds found in the present have been reported to have significant pharmacological, medicinal, and cosmetic applications, as they have been found to possess antimicrobial and antiseptic activity, anti-carcinogenic potential, neuroprotective activity, anti-obese activity, anti-diabetic effect, and analgesic activity as well as provide cardiovascular protection [8,11]. Another significant application is in the food industry, as the bioactive compounds of ginger can provide oxidative and storage stability, sensorial properties, preservation, oxidative resistance, and anti-bacterial activity in consuming products [7]. As well, another notable factor is its application in agriculture for the control of plant diseases which minimizes simultaneously the possible negative effects on the environment, animals, and human health [9,10].

## 3. Materials and Methods

### 3.1. Materials

Ethyl acetate (99.5%) was obtained from NEON comercial (Suzano, SP, Brazil), ethanol (99.5%) from ACS CIENTIFICA (Sumare, SP, Brazil), hexane (98.5%) from exodo cientifica (Sumare, SP, Brazil), CO_2_ (99.95%) and propane (99.5%) from White Martins S.A. (Curitiba, PR, Brazil), Folin-Ciocalteu’s reagent from CARLOS ERBA REAGENTS (Val-de-Reuil, France), Sodium carbonate anhydrous (>99.5%) from Fluka (Buchs, Switzerland), 1,1-Diphenyl-2-picrylhydrazyl Free Radical (DPPH) from TCI EUROPE N.V. (Zwijndrecht, Belgium), and gallic acid from Sigma Aldrich (Darmstadt, Germany). The Soxhlet extractor was purchased from Qualividros (Passos, MG, Brazil).

### 3.2. Sample Preparation

Very fresh *Zingiber officinale* Roscoe rhizomes were purchased from the local market at Curitiba, Brazil. After being transferred to the laboratory, the rhizomes were washed carefully with water and cut into smaller sized pieces to dry evenly. After being dried at 30 °C with air circulation until the moisture content became less than 10% (Table 8), the samples were grounded in a blender and stored in plastic bags until use.

### 3.3. Soxhlet Extraction

Soxhlet extraction was performed according to the AOAC [48] method. Briefly, 5 g of biomass and 150 mL of solvent were used in each extraction. The total duration was 6 h, using different temperatures depending on the solvent (Table 1). Specifically, the extractions took place at each solvent’s boiling point. The solvents used were ethyl acetate, ethanol, hexane, and water and, in each case, the procedure was repeated three times. A rotary vacuum evaporator (IKA RV 10 combined with IKA HB 10-IKA, Campinas, SP, Brazil) was used to concentrate the Soxhlet extracts, and they were then dried in an air circulation oven at 40 °C for about 24 h. The extraction yields were calculated with the Equation (1):

Yield (%) = [mass of dried extract (g)/initial mass of biomass (g)] × 100(1)

### 3.4. Sub- and Supercritical Extraction

Sub- and supercritical extractions were performed at an extraction unit (inner volume 80 cm^3^, length 16 cm and ∅ = 2.52 cm) which consisted of the extractor and a syringe pump. Additionally, the extraction system was equipped with pressure and temperature sensors in order to monitor the conditions. A thermostatic bath was attached to the system to better control the temperature. Furthermore, a needle valve controlled the flow inside the extractor, which was directly proportional to the pressure. The extractor was loaded with 10–15 g of biomass. Ιn the cases a co-solvent was used, its mass was 20–25 g. The temperature was set at 60 °C. For the scPropane extraction, the pressure was set at 100 bar, and for the scCO_2_ and scCO_2_ +EtOH extraction the pressure was set at 150 bar. The CO_2_ or the propane was loaded in the vessel until the desired pressure was achieved. In the beginning of each extraction process, a 30 min static extraction was performed, and after that a dynamic extraction was carried out with a flow rate of approximately 1–2 mL min^−1^. The dynamic extraction ended when no more extract was collected in the sampling tubes. The extraction yields were calculated by the Equation (1).

Extraction of the used primary biomass was also performed to evaluate the possibility of obtaining compounds of interest even with secondary biomass. Specifically, biomass prior used in scCO_2_ extraction was extracted a second time using scCO_2_ + EtOH. Moreover, a multistep approach was performed for the primary biomass that was used in scCO_2_ + EtOH, which then was extracted again twice with scCO_2_ + EtOH.

### 3.5. Total Phenolic Contents and Antioxidant Capacity

The total phenolic contents were determined using the Folin-Ciocalteu method as described by Box, 1983 [49]. Briefly, 5 mL of 10 μg mL^−1^ extract diluted in ethanol was added in glass bottles with 0.25 mL of Folin-Ciocalteu Reagent. After gentle stirring and 2 min of waiting, 0.75 mL of Na_2_CO_3_ 200 g L^−1^ was added and the solution was left in the dark for 1 h. The absorbance was measured at 765 nm in a Hitachi U2000 Spectrophotometer (Hitachi, Ltd., Santa Clara, CA, USA). The results were calculated using a calibration curve of gallic acid (0.5–10 mg L^−1^), and expressed as mg of gallic acid equivalents per 100 g of sample.

The free radical quenching ability of the extracts was determined using the DPPH reagent, as described by Mensor et al. [50]. Briefly, 2.5 mL of extract diluted in ethanol in concentrations ranging from 5 to 250 μg mL^−1^ (apart from the extract from Soxhlet that water was used as solvent, where the concentrations were ranging between 5 and 1000 μg mL^−1^, because of the low antioxidant capacity it presented) and DPPH in a final concentration of 0.3 mM were mixed. After 30 min in the dark, the absorbance was measured at 518 nm (Hitachi U2000 Spectrophotometer). The antioxidant capacity (AC) was calculated using the equation:

AC (%) = 100 − {[(abs_sample_ − abs_blank_) × 100]/abs_control_} (2)

where abs_sample_ is the absorbance of the solution consisting of the extract, DPPH, and ethanol, abs_blank_ the absorbance of the extract and ethanol, and abs_control_ the absorbance of DPPH and ethanol.

After the results were plotted, the concentration (μg mL^−1^) of each extract needed to inhibit 50% (IC_50_) of radicals’ production was calculated by linear regression.

### 3.6. Statistics

Significant differences in the yield percentages, total phenolic contents and IC_50_ values were assessed by post-hoc multiple comparison tests (Bonferroni test, *p* < 0.05, ANOVA) using the IBM SPSS statistics Inc. (Version: 28.0.1.0 (142)) software package.

### 3.7. GC-MS Analysis

Analysis of the samples (injection volume 1 μL) were performed using two GC-MS systems. The first system was composed of an Agilent 6890 Series GC system and an Agilent 5973 Network mass selective detector (MSD) (Agilent Technologies, Santa Clara, CA, USA). The second system was an Agilent 5975B inert MSD integrated to an Agilent 6890N Network GC (Agilent Technologies, Santa Clara, CA, USA). More information about the analytical columns, the temperature programs, and the conditions adopted in the MSD are reported in Supplementary Materials. Enhanced Data Analysis software was used for the analysis of the chromatograms, and NIST MS Search 2.0 software was used for compound identification.

## 4. Conclusions

In this work, compressed solvents technology was investigated for the extraction of high value products from *Zingiber officinale* Roscoe. Soxhlet extraction was used in preliminary experiments for the selection of the most appropriate co-solvent for scCO_2_. Using Soxhlet extraction, the highest yields were observed when water and ethanol were used, i.e., 17.93% and 17.7%, respectively. Ethanol was selected as the co-solvent for the supercritical extraction due to its high yield percentage, total phenolic content, and antioxidant capacity. When compressed solvents were used, the most efficient was scCO_2_ + ethanol, reaching about 6% extraction yield. High antioxidant activity was also observed for the ginger extracts in all cases.

The proposed procedure for the extraction of the secondary biomass used CO_2_ and ethanol as co-solvents, resulting in high extraction yields (reaching 6.42%) and an accelerated extraction time. Except the high extraction yields, another advantage of the reusage of the biomass is that, even after two extractions, high value bioactive compounds, such as zingerone and gingerol, were detected, and the extracts presented high antioxidant capacity with IC_50_ values up to 132.39 μg mL^−1^.

The extracts obtained with all the tested methods presented similar chemical compositions, and the most abundant substances were a-zingiberene, β-sesquiphellandrene, a-farnesene, β-bisabolene, zingerone, gingerol, a-curcumene, and γ-muurolene. All these compounds present beneficial properties and can have real applications in various sectors, such as the food and pharmaceutical industries. After the first reuse, high value compounds were identified as well, similar to those of the primary biomass. Taking into consideration the high yield percentages and the antioxidant capacity, as well as the chemical composition, it is safe to conclude that the addition of ethanol as a co-solvent in the scCO_2_ extraction had a positive effect both in the extraction of the primary biomass and in the secondary biomass extraction. Based on the results, the reuse of a secondary biomass (raw material) presents high significance. Indeed, the maximum utilization of biomass can contribute to the achievement of the goals of circular economy. Furthermore, although not tested in this work, the residual biomass after the secondary extraction could potentially be used as a raw fertilizing/pest repelling compound, enhancing the soils that are employed in agriculture.

## Figures and Tables

**Table 1:** Experimental conditions and extraction yields of *Zingiber officinale* Roscoe acquired under 6 h of Soxhlet extraction using different solvents.

Solvents	Polarity *	Boiling Point (°C) **	Type of Biomass	Yield (%) ± SD
Ethyl acetate	4.3	77.0	Primary	8.28 ± 0.48 ^abc^
Ethanol	5.2	78.5	Primary	17.70 ± 1.78 ^ad^
Hexane	0.0	63.9	Primary	4.82 ± 0.23 ^bde^
Water	10.2	100.5	Primary	17.93 ± 6.67 ^ce^

SD: standard deviation, ^abcde^: values in each column that share the same letter are significantly different from each other (One-way Anova, *p* < 0.05),* [22], ** [23].

**Table 2:** Antioxidant capacity and total phenolic content (mg of gallic acid equivalents (GAE) per 100 g of sample) of primary biomass of *Zingiber officinale* Roscoe extracts acquired under Soxhlet extraction using different solvents.

Solvents	IC_50_ (μg mL^−1^) ± SD	Total Phenolic Content (mg GAE 100 g^−1^) ± SD
Ethyl Acetate	150.50 ± 6.06 ^a^	1.96 ± 0.10 ^ab^
Ethanol	191.16 ± 10.58 ^b^	7.04 ± 0.79 ^acd^
Hexane	201.05 ± 7.22 ^c^	0.96 ± 0.16 ^c^
Water	758.29 ± 29.59 ^abc^	0.83 ± 0.42 ^bd^

SD: standard deviation, ^abcd^: values in each column that share the same letter are significantly different from each other (One-way Anova, *p* < 0.05).

**Table 3:** Extraction yields of *Zingiber officinale* Roscoe acquired under subcritical and supercritical extraction using different extraction conditions.

Solvents	Temperature (°C)	Pressure (Bar)	Type of Biomass	Time of Extraction (Min)	Yield (%) ± SD
Propane	60	100	Primary	130	2.06 ± 0.01 ^abcde^
CO_2_	60	150	Primary	140	1.54 ± 0.01 ^afghi^
CO_2_ + EtOH	60	150	Primary	60	6.06 ± 0.01 ^bfjkl^
CO_2_ + EtOH	60	150	Secondary (Used once in scCO_2_)	40	6.42 ± 0.01 ^cgjmn^
CO_2_ + EtOH	60	150	Secondary (Used once in scCO_2_ + EtOH)	40	3.20 ± 0.01 ^dhkmo^
CO_2_ + EtOH	60	150	Secondary (Used twice in scCO_2_ + EtOH)	40	1.23 ± 0.01 ^eilno^

SD: standard deviation, ^a–o^: values in each column that share the same letter are significantly different from each other (One-way Anova, *p* < 0.05).

**Table 4:** Total phenolic content of *Zingiber officinale* Roscoe extracts acquired under different extraction conditions (mg GAE 100 g^−1^).

Solvents	Extraction Condition	Type of Biomass	Total Phenolic Content (mg GAE 100 g^−1^) ± SD
Propane	100 bar/60 °C	Primary	1.76 ± 0.42
CO_2_	150 bar/60 °C	Primary	1.65 ± 0.27
CO_2_ + EtOH	150 bar/60 °C	Primary	1.87 ± 0.18
CO_2_ + EtOH	150 bar/60 °C	Secondary (Used once in scCO_2_)	1.47 ± 0.04 ^a^
CO_2_ + EtOH	150 bar/60 °C	Secondary (Used once in scCO_2_ + EtOH)	1.94 ± 0.08 ^a^
CO_2_ + EtOH	150 bar/60 °C	Secondary (Used twice in scCO_2_ + EtOH)	1.74 ± 0.05

SD: standard deviation, ^a^: values in each column that share the same letter are significantly different from each other (One-way Anova, *p* < 0.05).

**Table 5:** Antioxidant capacity of *Zingiber officinale* Roscoe extracts acquired under different extraction conditions.

Solvents	Extraction Condition	Type of Biomass	IC_50_ (μg mL^−1^) ± SD
Propane	100 bar/60 °C	Primary	153.14 ± 0.21 ^abcde^
CO_2_	150 bar/60 °C	Primary	169.74 ± 5.16 ^afghi^
CO_2_ + EtOH	150 bar/60 °C	Primary	125.12 ± 3.32 ^bf^
CO_2_ + EtOH	150 bar/60 °C	Secondary (Used once in scCO_2_)	131.83 ± 6.95 ^cg^
CO_2_ + EtOH	150 bar/60 °C	Secondary (Used once in scCO_2_ + EtOH)	132.39 ± 1.91 ^dh^
CO_2_ + EtOH	150 bar/60 °C	Secondary (Used twice in scCO_2_ + EtOH)	127.54 ± 3.32 ^ei^

SD: standard deviation, ^a–i^: values in each column that share the same letter are significantly different from each other (One-way Anova, *p* < 0.05).

**Table 6:** Chemical composition of ginger extracts (Type of biomass: Primary) using different extraction methods (+:presence/% of total area).

Compounds	Soxhlet/EtOH	scPropane	scCO_2_	scCO_2_ + EtOH
**GC-MS (System 1)**				
Hexanal	+(0.634%)	+(0.736%)	+(0.134%)	+(1.054%)
Decanal	+(1.830%)	+(1.225%)	+(0.934%)	+(1.512%)
α-Terpineol		+(0.550%)		+(0.789%)
cis-Geraniol		+(0.471%)	+(1.269%)	+(0.851%)
Geranial		+(1.358%)	+(0.678%)	
Geraniol acetate		+(0.390%)	+(1.569%)	
β-Bisabolene		+(1.988%)	+(2.002%)	+(5.3147%)
a-Farnesene	+(7.397%)	+(6.919%)	+(7.318%)	+(8.003%)
a-Zingiberene	+(45.072%)	+(57.398%)	+(52.105%)	+(46.355%)
β-Sesquiphellandrene	+(14.685%)	+(13.542%)	+(15.905%)	+(17.111%)
γ-Cadinene		+(2.486%)	+(2.838%)	+(3.094%)
Valencene		+(1.544%)	+(1.985%)	+(2.072%)
Zingerone	+(30.383%)	+(11.394%)	+(13.262%)	+(13.842%)
**GC-MS (System 2)**				
Cedrene	+(0.885%)	+(2.835%)		+(1.285%)
γ-Patchoulene	+(1.325%)		+(1.984%)	
a-Curcumene	+(26.152%)	+(28.745%)	+(25.244%)	+(22.418%)
Germacrene D		+(2.568%)		
γ-Muurolene	+(22.562%)		+(20.682%)	+(16.854%)
α-Bergamotene	+(1.862%)			
α-Eudesmol	+(1.859%)	+(5.624%)	+(2.856%)	+(3.145%)
Gingerol	+(40.152%)		+(30.658%)	+(29.745%)
Borneol		+(3.452%)		
(+)-Cycloisosativene		+(3.526%)		
Nerolidyl acetate		+(5.125%)	+(3.256%)	+(2.256%)
γ-Elemene		+(6.514%)		+(3.652%)
β-Farnesene	+(5.203%)	+(7.418%)		
(−)-allo-Aromadendrene		+(3.215%)	+(2.562%)	+(1.523%)
α-Himachalene		+(4.158%)		+(3.052%)
Elemol		+(3.103%)		
γ-Gurjunene		+(8.774%)		+(6.135%)
γ-Eudesmol		+(5.263%)	+(2.365%)	+(4.090%)
Cubenol		+(2.259%)		
Cedr-8-en-13-ol		+(4.156%)	+(4.526%)	
Cubenol		+(3.265%)	+(3.269%)	
Urs-12-en-28-ol			+(2.598%)	+(2.589%)
8-Isopropenyl-1,5-dimethyl-1,5-cyclodecadiene				+(3.256%)

**Table 7:** Chemical composition of ginger extracts (Type of biomass: Secondary) using different and sequential extraction methods (+: presence/% of total area).

Compounds	scCO_2_ + EtOH after scCO_2_	scCO_2_ + EtOH after One scCO_2_+ EtOH	scCO_2_ + EtOH after Two scCO_2_ + EtOH
**GC-MS (System 1)**			
Hexanal	+(0.720%)		
a-Farnesene	+(12.464%)	+ (9.598%)	
a-Zingiberene	+(48.558%)	+(37.036%)	
β- Sesquiphellandrene	+(8.409%)	+(5.637%)	
γ-Cadinene	+(16.834%)	+(14.008%)	
Zingerone	+(13.015%)	+(33.721%)	+(100%)
**GC-MS (System 2)**			
Cedrene	+(1.526%)		
a-Curcumene	+(25.032%)	+(24.195%)	
γ-Muurolene	+(14.012%)	+(13.589%)	
α-Bergamotene	+(5.141%)	+(6.524%)	
α-Eudesmol	+(2.154%)	+(3.528%)	
Gingerol	+(52.135%)	+(52.164%)	+(100%)

**Table 8:** Moisture content of *Zingiber officinale* Roscoe before and after drying.

Sample	Average Moisture Content (%) ± SD
Fresh	89.05 ± 1.20
Dried	8.96 ± 0.22

SD: standard deviation.

## Data Availability

Data are contained within the article.

## Supplementary Materials

The following supporting information can be downloaded at: https://www.mdpi.com/article/10.3390/molecules29040871/s1, Supplementary Materials S1. GC-MS Analysis, Figure S1: Antioxidant capacity (%) of *Zingiber officinale* Roscoe extracts (μg mL^−1^) acquired under Soxhlet extraction using (a) ethyl acetate, (b) ethanol, (c) hexane and (d) water as solvents. Figure S2: Antioxidant capacity (%) of *Zingiber officinale* Roscoe extracts (μg mL^−1^) acquired under (a) scPropane extraction, (b) scCO_2_ and (c) scCO_2_ +EtOH. Figure S3: Antioxidant capacity (%) of *Zingiber officinale* Roscoe extracts (μg mL^−1^) acquired under scCO_2_ +EtOH, with biomass previously used at (a) scCO_2_ once, (b) scCO_2_ +EtOH once and (c) scCO_2_ +EtOH twice.
