# Supplementary material for: Extraction of High Value Products from Zingiber officinale Roscoe (Ginger) and Utilization of Residual Biomass
Source: Molecules. 2024 Feb 16;29(4):871. doi: 10.3390/molecules29040871 (PMC10893072; doi:10.3390/molecules29040871)
Supplement: Supplementary file 1 [file molecules-29-00871-s001.zip › molecules-2826179-supplementary.pdf]

Supplementary Materials

Extraction of High Value Products from *Zingiber Officinale* Roscoe (Ginger) and Utilization of Residual Biomass

Alexandra Spyrou [1], Marcelle G. F. Batista [2], Marcos L. Corazza [2], Maria Papadaki [3,*] and Maria Antonopoulou [1,*]

[1] Department of Sustainable Agriculture, University of Patras, Seferi 2, GR30131 Agrinio, Greece; spyrou.a@upatras.gr (A.S.)
[2] Department of Chemical Engineering, Federal University of Parana Curitiba CEP 81531-990, PR, Brazil; marcelleguth@ufpr.br (M.G.F.B.); corazza@ufpr.br (M.L.C.)
[3] Department of Agriculture, Nea Ktiria, University of Patras, GR30200 Messolonghi, Greece
* Correspondence: marpapadaki@upatras.gr (M.P.); mantonop@upatras.gr (M.A.); Tel.: +30-26310-58428 (M.P.); +30-26410-74114 (M.A.)

Supplementary Materials S1. GC-MS Analysis

Using the first GC-MS system the analysis was performed on a Vocol™ fused silica analytical column from Supelco (30 m length, 0.25 mm diameter and 1.5 μm film thickness). The sample was injected in split mode and ratio 1:5 using an HP 6890 series injector (temperature =250 °C). The temperature program was: 50 °C (2 min), followed by ramp of 10° C/min, up to a final temperature of 240 °C (23 min). The duration of each run was 44 min.

An Agilent HP-5MS (30 m length, 250 μm diameter and 0.25 μm film thickness) analytical column was used for the analysis by the second GC-MS system. The sample was injected in splitless mode (temperature=300°C). The column temperature program was: 50 °C (2 min) followed by ramp of 10° C/min, up to a final temperature of 280 °C (15 min). The total run was 40 min.

In both systems, the MSD detectors were operated under the following conditions: ion source temperature=230 °C, quadrupole temperature= 150 °C and transfer line temperature=280 °C. The scan mode analysis in the range of m/z 50.00-600.00 was selected.

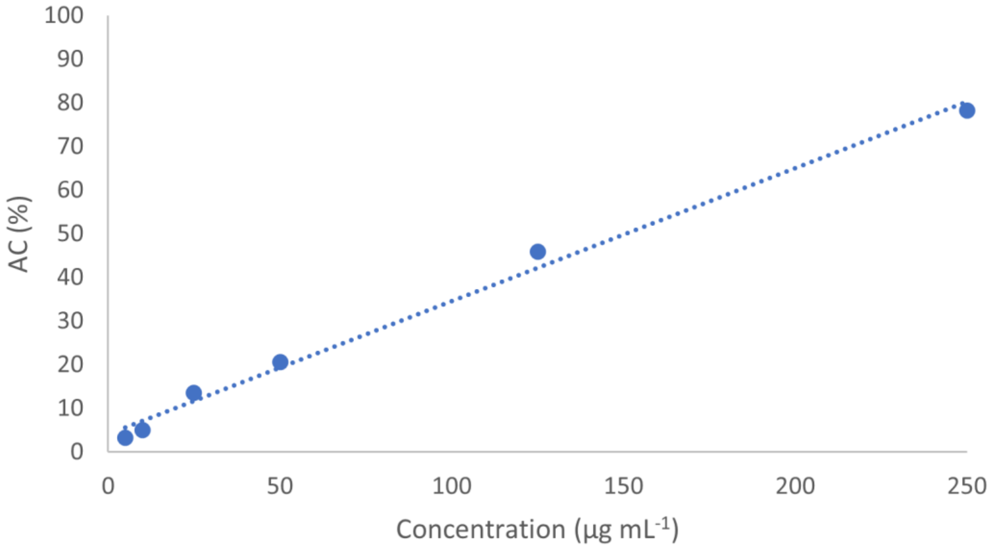
100
90
80
70
60
50
40
30
20
10
0
AC (%)
0
50
100
150
200
250
Concentration (μg mL-1)

(a)

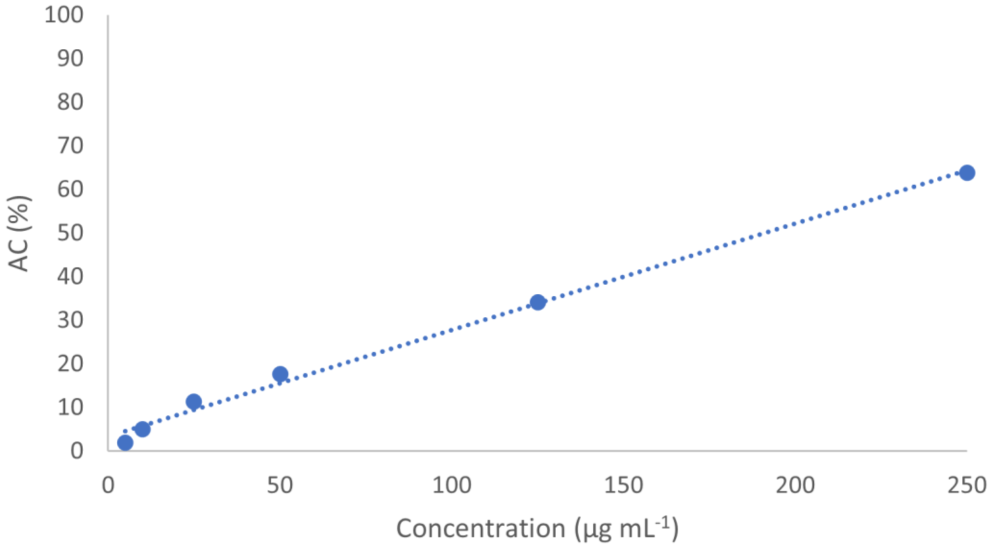
100
90
80
70
60
50
40
30
20
10
0
AC (%)
0
50
100
150
200
250
Concentration (μg mL-1)

(b)

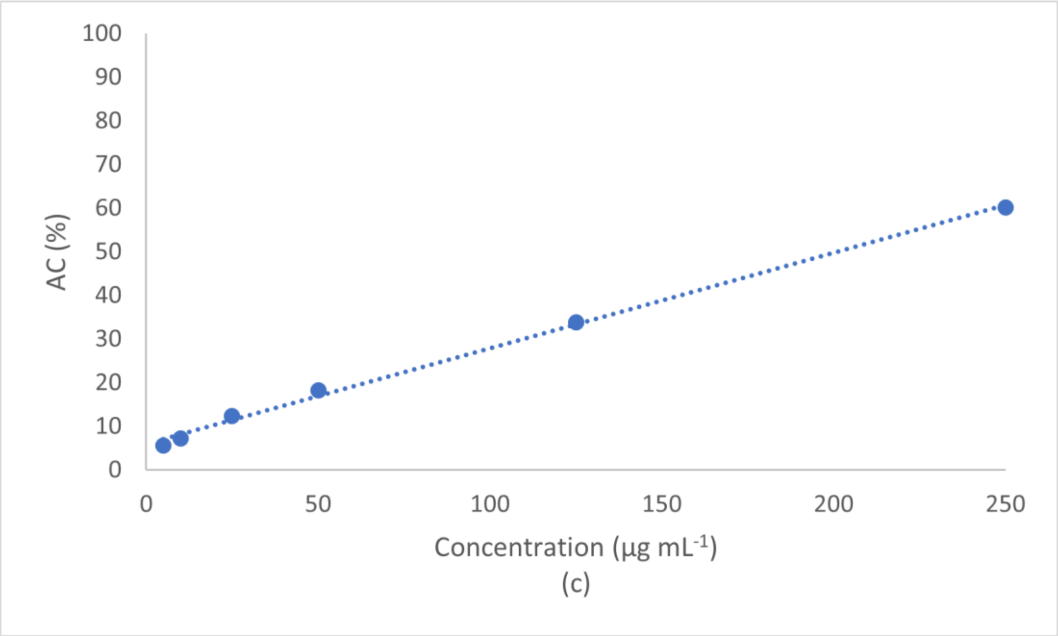

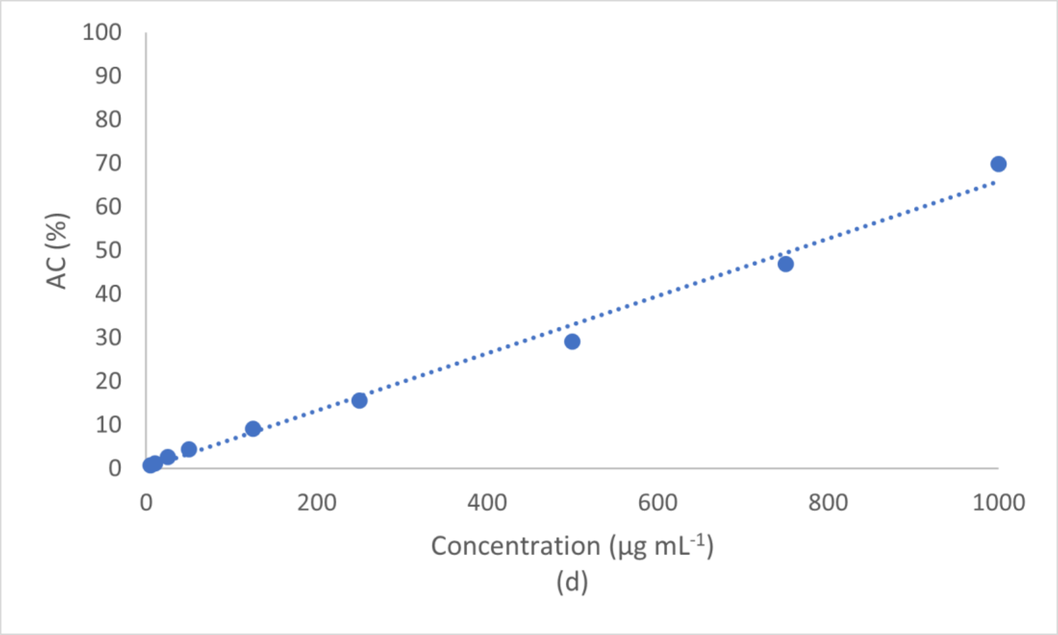

Figure S1: Antioxidant capacity (%) of *Zingiber officinale* Roscoe extracts (μg mL^{-1}) acquired under Soxhlet extraction using a) ethyl acetate, b) ethanol, c) hexane and d) water as solvents.

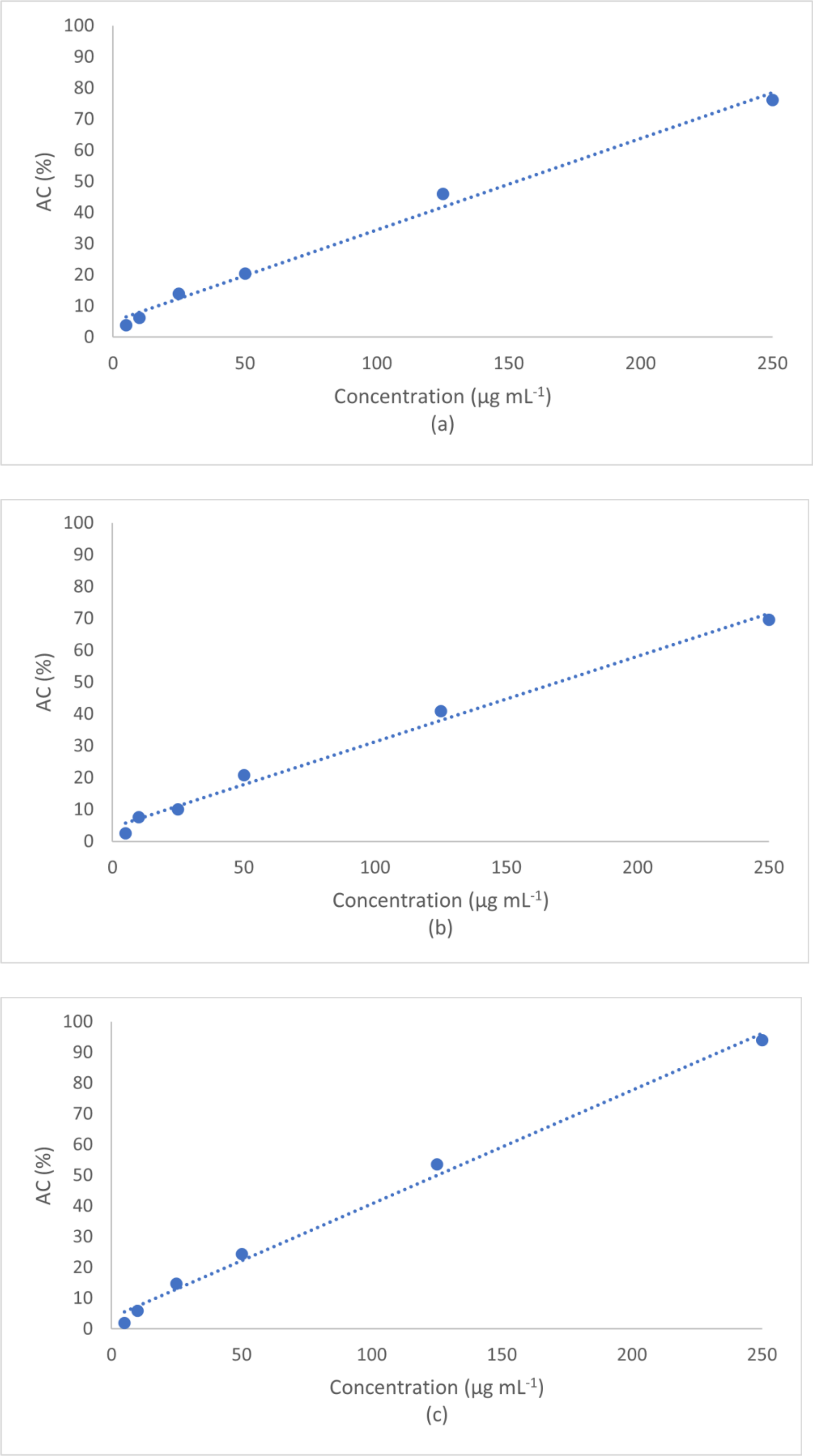

Figure S2: Antioxidant capacity (%) of *Zingiber officinale* Roscoe extracts (μg mL^{-1}) acquired under a) scPropane extraction, b) $scCO_2$ and c) $scCO_2$+EtOH.

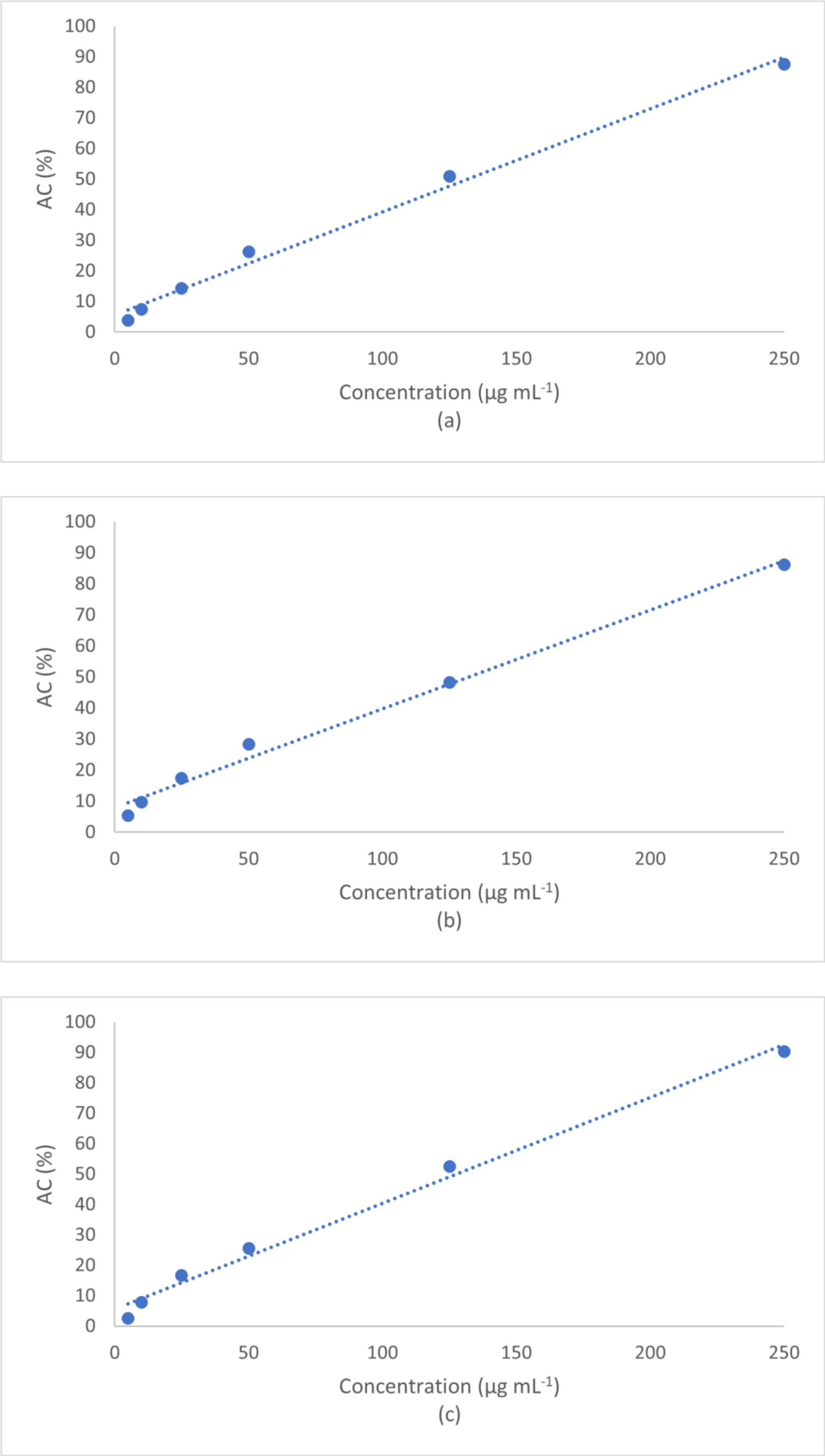

Figure S3: Antioxidant capacity (%) of *Zingiber officinale* Roscoe extracts (μg mL^{-1}) acquired under $scCO_2$+EtOH, with secondary biomass, previously used at a) $scCO_2$ once, b) $scCO_2$+EtOH once and c) $scCO_2$+EtOH twice.
